# Enhanced Production of Xylitol from Corncob by *Pachysolen tannophilus* Using Response Surface Methodology

**DOI:** 10.1155/2013/514676

**Published:** 2013-06-19

**Authors:** S. Ramesh, R. Muthuvelayudham, R. Rajesh Kannan, T. Viruthagiri

**Affiliations:** Department of Chemical Engineering, Annamalai University, Annamalainagar 608002, Tamil Nadu, India

## Abstract

Optimization of the culture medium and process variables for xylitol production using corncob hemicellulose hydrolysate by *Pachysolen tannophilus* (MTTC 1077) was performed with statistical methodology based on experimental designs. The screening of nine nutrients for their influence on xylitol production was achieved using a Plackett-Burman design. Peptone, xylose, MgSO_4_·7H_2_O, and yeast extract were selected based on their positive influence on xylitol production. The selected components were optimized with Box-Behnken design using response surface methodology (RSM). The optimum levels (g/L) were peptone: 6.03, xylose: 10.62, MgSO_4_·7H_2_O: 1.39, yeast extract: 4.66. The influence of various process variables on the xylitol production was evaluated. The optimal levels of these variables were quantified by the central composite design using RSM, for establishment of a significant mathematical model with a coefficient determination of *R* ^2^ = 0.91. The validation experimental was consistent with the prediction model. The optimum levels of process variables were temperature (36.56°C), pH (7.27), substrate concentration (3.55 g/L), inoculum size (3.69 mL), and agitation speed (194.44 rpm). These conditions were validated experimentally which revealed an enhanced xylitol yield of 0.80 g/g.

## 1. Introduction 

Lignocellulosic materials represent an abundant and inexpensive source of sugars and can be microbiologically converted to industrial products. Xylitol (C_5_H_12_O_5_), a sugar alcohol obtained from xylose, is generated during the metabolism of carbohydrates in animals and humans. Its concentration in human blood varies from 0.03 to 0.06 mg/100 mL [1]. Xylitol was present in fruits and vegetables [2], at low concentration, which makes its production from these sources economically unfeasible [3]. As a sweetener, xylitol is a substitute for conventional sugars [4]. Its sweetening power was comparable to that of sucrose and is higher than that of sorbitol and mannitol [5]. Furthermore, xylitol has anticariogenic properties. Because it is not consumed by *streptococcus mutans*, xylitol prevents the formation of acids that attack tooth enamel [6]. In addition to reducing dental caries, xylitol also promotes tooth enamel remineralization by reversing small lesions. This happens because, when in contact with xylitol, the saliva seems to be favorably influenced; the chemical composition of xylitol induces the calcium ions and phosphate [7]. For these characteristics, xylitol was a feed stock of great interest to food, odontological, and pharmaceutical industries [1].

Currently, xylitol is produced by chemical hydrogenation using nickel as a catalyst [8]. However it was expensive and it requires several steps of xylose purification before the chemical reaction [4, 9, 10]. Xylitol production through bioconversion has been proposed as for utilizing microorganism such as yeast, bacteria, and fungi [11, 12]. Among these, yeast has shown to possess desirable properties for xylitol production [13, 14]. Therefore, for the present study, yeast strain *Pachysolen tannophilus* was selected for xylitol production. Furthermore studies have shown that nutritional factors including sources of carbon and nitrogen can influence xylitol production [15]. 

Corncob is a large volume solid waste for using sweet corn processing industry in India. They are currently used as animal feed or returned to the harvested field for land application [16]. Corncob contains approximately over 40% of the dry matter in residues [17] and thus has value has a raw material for production of xylose, xylitol, arabinose, xylobiose, and xylo oligosaccharides. The hemicelluloses fraction in corncob can be easily hydrolysed to constituent carbohydrates. These carbohydrates mainly consist of the xylose and other minor pentose [18–20]. Among various agricultural wastes, corncob was regarded as promising agricultural resources for microbial xylitol production.

In microbial production of xylitol from corncob, the cobs were first hydrolysed to produce from hemicelluloses by acid hydrolysis and the corncob hydrolysate is then used as the medium for xylitol production. The bioconversion of xylitol is influenced by the concentration of various ingredients in culture medium. So their optimization study was very important. This study also investigates the effect of process variables such as pH, temperature, substrate concentration, inoculum size, and agitation speed on xylitol yield. Response surface methodology (RSM) is a mathematical and statistical analysis, which is useful for the modeling and analysis problems that the response of interest is influenced by several variables [21]. RSM was utilized extensively for optimizing different biotechnological process [22, 23]. 

In the present study, the screening and optimization of medium composition and process variables for xylitol production by* Pachysolen tannophilus* using Plackett-Burman and RSM were reported. The Plackett-Burman screening design was applied for knowing the most significant nutrients enhancing xylitol production. Then, Box-Behnken design and central composite design (CCD) were applied to determine the optimum level of significant nutrients and process variables, respectively.

## 2. Materials and Methods 

### 2.1. Microorganisms and Maintenance

The yeast strain *Pachysolen tannophilus* (MTCC 1077) was collected from Microbial Type Culture Collection and Gene bank, Chandigarh. The lyophilized stock cultures were maintained at 4°C on culture medium supplemented with 20 g of agar. The medium composition (g/L) was compressed of the following: malt extract: 3.0; yeast extract: 3.0; peptone: 5.0; glucose: 10.0 at pH: 7. It was subcultured every thirty days to maintain viability.

### 2.2. Size Reduction

Corncob was collected from perambalur farms, Tamil Nadu, India, and was dried in sunlight for 2 days, crushed, and sieved for different mesh size ranging from 0.45 mm to 0.9 mm (20–40 mesh) and used for further studies. The composition of the corncob (g/L): xylose: 28.7, glucose: 5.4, arabinose: 3.7, cellobiose: 0.5, galactose: 0.7, mannose: 0.4, acetic acid: 2, furfural: 0.8, hydroxymethyl furfural: 0.2 was used for xylitol production. 

### 2.3. Acid Hydrolysis

The pretreatment was carried out in 500 mL glass flasks. 2 g of corncobs at a solid loading of 10% (w/w) was mixed with dilute sulfuric acid (0.1% (w/v)) and pretreated in an autoclave at 120°C with residence time of 1 hour. The liquid fraction was separated by filtration and the unhydrolysed solid residue was washed with warm water (60°C). The filtrate and wash liquid were pooled together.

### 2.4. Detoxification

Hemicellulose acid hydrolysate was heated at 100°C for 15 min to reduce the volatile components. The hydrolysate was overlined with solid Ca(OH)_2_ up to pH 10, in combination with 0.1% sodium sulfite, and filtered to remove the insoluble materials. The filtrate was adjusted to pH 7 with H_2_SO_4_. The water phase was treated with activated charcoal.

### 2.5. Activated Charcoal Treatment

Activated charcoal treatment was an efficient and economic method of reduction in the amount of phenolic compounds, acetic acid, aromatic compounds, furfural, and hydroxymethylfurfural normally found in hemicellulosic hydrolysates. After centrifugation, the solutions were mixed with powdered charcoal at 5% (w/v) for 30 and stirred (100 rpm) at 30°C. The liquor was recovered by filtration, chemically characterized, and used for culture media.

### 2.6. Fermentation

Fermentation was carried out in 250 mL Erlenmeyer flasks with 100 mL of pretreated corncob hemicelluloses hydrolysate is adjusted to pH 7 with 2 M H_2_SO_4_ or 3 M NaOH and supplemented with different nutrients concentration for tests according to the selected factorial design, were used for fermentation medium and sterilized at 120°C for 20 mins. After cooling the flasks were inoculated with 1 mL of grown culture broth. The flasks were maintained at 30°C for agitation at 200 rpm for 48 hours. After the optimization of medium composition, the fermentation was carried out with different parameter levels (Table 5) with the optimized media for tests according to the selected factorial design. During the preliminary screening, the experiments were carried out for 5 days and the maximum production was obtained in 48 hours. Hence experiments were carried out for 48 hours.

### 2.7. Analytical Methods

Sugar and sugar alcohol in the culture broth were measured by high-performance liquid chromatography (HPLC), model LC-10-AD (Shimadzu, Tokyo, Japan) equipped with a refractive index (RI) detector. The chromatography column used was a Aminex HPX-87H (300 × 7.8 mm) column at 80°C with 5 mm H_2_SO_4_ as mobile phase at a flow rate of 0.4 mL/min, and the injected sample volume was 20 *μ*L.

### 2.8. Optimization of Xylitol Production: Design of Experiment (DOE)

The RSM has several classes of designs, with its own properties and characteristics. Central composite design (CCD), Box-Behnken design, and three-level factorial design are the most popular designs applied by the researchers. A prior knowledge with understanding of the related bioprocesses is necessary for a realistic modeling approach.

### 2.9. Plackett-Burman Experimental Design

It assumes that there are no interactions between the different variables in the range under consideration. A linear approach is considered to be sufficient for screening. Plackett-Burman experimental design is a fractional factorial design and the main effects of such a design may be simply calculated as the difference between the average of measurements made at the high level (+1) of the factor and the average of measurements at the low level (−1). To determine which variables significantly affect xylitol production, Plackett-Burman design is used. Nine variables were screened in 12 experimental runs (Table 1), and insignificant ones are eliminated in order to obtain a smaller, manageable set of factors. The low level (−1) and high level (+1) of each factor (−1, +1) were listed as follows (g/L): K_2_HPO_4_ (6.6, 7), yeast extract (1.5, 5), peptone (2, 5), KH_2_PO_4_ (1.2, 3.6), xylose (9.8, 10.2), (NH_4_)_2_SO_4_ (1, 4), MgSO_4_·7H_2_O (0.7, 1.3), malt (2.8, 3.2), and glucose (9.8, 10.2), and they were coded with *A*, *B*, *C*, *D*, *E*, *F*, *G*, *H*, *I*, respectively. The statistical software package “Minitab 16” is used for analyzing the experimental data. Once the critical factors are identified through the screening, the Box-Behnken design was used to obtain a quadratic model after the central composite design (CCD) was used to optimize the process variables and obtain a quadratic model.

The Box-Behnken design and CCD was used to study the effects of the variables towards their responses and subsequently in the optimization studies. This method was suitable for fitting a quadratic surface, and it helps to optimize the effective parameters with a minimum number of experiments, as well as to analyze the interaction between the parameters. In order to determine the relationship between the factors and response variables, the data collected were analyzed in statistical manner. A regression design was employed to model a response as a mathematical function (either known or empirical) for few continuous factors, and good model parameter estimates are desired [21].

The coded values of the process parameters are determined by the following equation:

(1) $$\begin{array}{c} x_{i}=\frac{X_{i}-X_{0}}{\Delta x}, \end{array}$$

where *x*_*i*_ is coded value of the *i*th variable, *X*_*i*_ is uncoded value of the *i*th test variable, and *X*_0_ is uncoded value of the *i*th test variable at center point. The regression analysis is performed to estimate the response function as a second-order polynomial:

(2) $$\begin{array}{c} Y=\beta_{0}+\sum_{i=1}^{K}\beta_{i}X_{i}+\sum_{i=1}^{K}\beta_{ii}X_{i}^{2}\sum_{i=1,\;i<j}^{K-1}\;\sum_{j=2}^{K}\beta_{ij}X_{i}X_{j}, \end{array}$$

where *Y* is the predicted response, *β*_0_ constant, and *β*_*i*_, *β*_*ii*_, *β*_*ij*_ are coefficients estimated from regression. They represent the linear, quadratic, and cross-products of *X*_*i*_ and *X*_*j*_ on response.

### 2.10. Model Fitting and Statistical Analysis

The regression and graphical analysis are carried out using Design-Expert software (version 7.1.5, Stat-Ease, Inc., Minneapolis, USA). The optimum values of the process variables were obtained from the regression equation. The adequacy of the models is further justified through analysis of variance (ANOVA). Lack-of-fit is a special diagnostic test for adequacy of a model that compares the pure error, based on the replicate measurements to the other lack of fit, based on the model performance [24]. *F* value, calculated as the ratio between the lack-of-fit mean square and the pure error mean square, is the statistic parameter used to determine whether the lack-of-fit is significant or not, at a significance level. The statistical model was validated with respect to xylitol production under the conditions predicted by the model in shake-flask level. Samples were drawn at the desired intervals and xylitol production was determined as described above.

## 3. Results and Discussions

**Figure 1:**
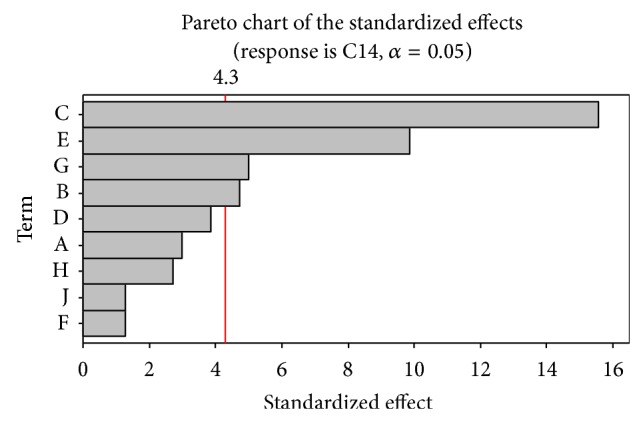
Pareto chart showing the effect of media components on xylitol production.

Plackett-Burman experiments (Table 1) showed a wide variation in xylitol production. This variation reflected the importance of optimization to attain higher productivity. From the Pareto chart shown in Figure 1 the variables, namely, peptone, xylose, MgSO_4_·7H_2_O, and yeast extract, were selected for further optimization to attain a maximum response.

The levels of factors and the effect of their interactions on xylitol production were determined by Box-Behnken design of RSM. The design matrix of experimental results by tests was planned according to the 29 full factorial designs. Twenty-nine experiments were performed at different combinations of the factors shown in Table 2, and the central point was repeated five times. The predicted and observed responses along with design matrix are presented in Table 3, and the results were analyzed by ANOVA. The second-order regression equation provided the levels of xylitol production as a function of peptone, xylose, MgSO_4_·7H_2_O, and yeast extract, which can be presented in terms of coded factors as in the following equation:

(3) Y=0.70+0.053A+0.018B+0.057C+0.054D +0.092AB−(2.500E−003)AC +(1.000E−002)AD+0.028BC+0.077BD −0.040CD−0.083A2−0.16B2−0.076C2−0.11D2,

where *Y* is the xylitol yield (g/g) and *A*, *B*, *C*, and *D* were peptone, xylose, MgSO_4_·7H_2_O, and yeast extract, respectively. ANOVA for the response surface was shown in Table 4. The model *F* value of 26.29 implies that the model is significant. There is only a 0.01% chance that a “Model *F*-value” this large could occur due to noise. Values of “prob > *F*” less than 0.05 indicate that model terms are significant. Values greater than 0.1 indicate that model terms are not significant. In the present work, linear terms of *A*, *C*, and  *D* and all the square effects of *A*, *B*, *C*, and *D* and the combination of *A*∗*B*, *B*∗*D*, and *C*∗*D* were significant for xylitol production. The coefficient of determination (*R* ^2^) for xylitol production was calculated as 0.9634, which is very close to 1 and can explain up to 96.00% variability of the response. The predicted *R* ^2^ value of 0.7898 was in reasonable agreement with the adjusted *R* ^2^ value of 0.9267. An adequate precision value greater than 4 is desirable. The adequate precision value of 16.010 indicates an adequate signal and suggests that the model can navigate the design space.

The above model can be used to predict the xylitol production within the limits of the experimental factors that the actual response values agree well with the predicted response values.

Experimental conditions for optimization of the process variables for xylitol yield were determined by CCD of RSM. Five process variables are assessed at 5 coded levels as shown in Table 5. The design matrix of experimental results by tests was planned according to the 50 full factorial designs, and the central point was repeated eight times. The predicted and observed responses along with design matrix are presented in Table 6 and the results were analyzed by ANOVA.

The second-order regression equation provided the levels of xylitol production as a function of temperature, substrate concentration, pH, agitation speed, and inoculums size, which can be presented in terms of coded factors as in the following equation:

(4) Y=0.79+0.025A+0.043B+0.049C +0.030D+0.038E−0.029AB−(4.063E−003)AC +0.018AD+(2.187E−003)AE−(9.688E−003)BC +0.014BD+(5.312E−003)BE+(1.562E−003)CD +(5.312E−003)CE−(3.125E−004)DE−0.040A2 −0.041B2−0.046C2−0.027D2−0.034E2,

where *Y* was the xylitol yield (g/g) and *A*, *B*, *C*, *D*, and *E* are temperature, substrate concentration, pH, agitation speed, and inoculums size, respectively. ANOVA for the response surface was shown in Table 7. The model *F* value of 15.58 implies that the model is significant. There is only a 0.01% chance that a “Model *F*-value” this large could occur due to noise. Values of “prob > *F*” less than 0.05 indicate that model terms are significant. Values greater than 0.1 indicate that model terms are not significant. In the present work, linear terms and all the square effects of *A*, *B*, *C*, *D*, and *E* and the combination of *A*∗*B* and *A*∗*D* were significant for xylitol production. The coefficient of determination (*R* ^2^) for xylitol production was calculated as 0.9148, which is very close to 1 and can explain up to 91.00% variability of the response. The predicted *R* ^2^ value of 0.6867 was in reasonable agreement with the adjusted *R* ^2^ value of 0.8561. An adequate precision value greater than 4 is desirable. The adequate precision value of 12.951 indicates an adequate signal and suggests that the model can navigate the design space.

**Figure 2:**
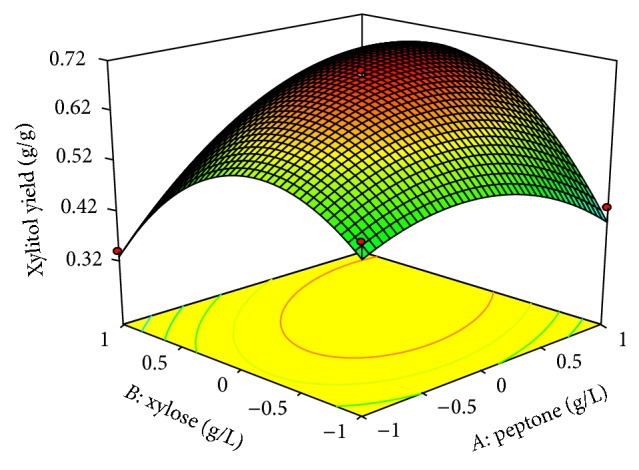
3D plot showing the effect of peptone and xylose on xylitol yield.

**Figure 3:**
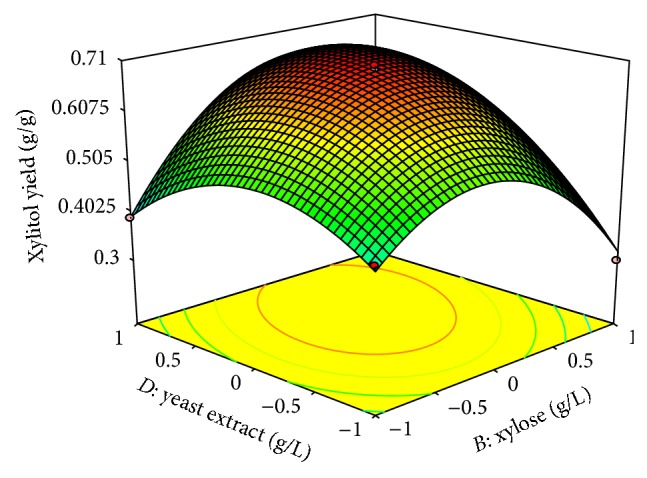
3D plot showing the effect of xylose and yeast extract on xylitol yield.

**Figure 4:**
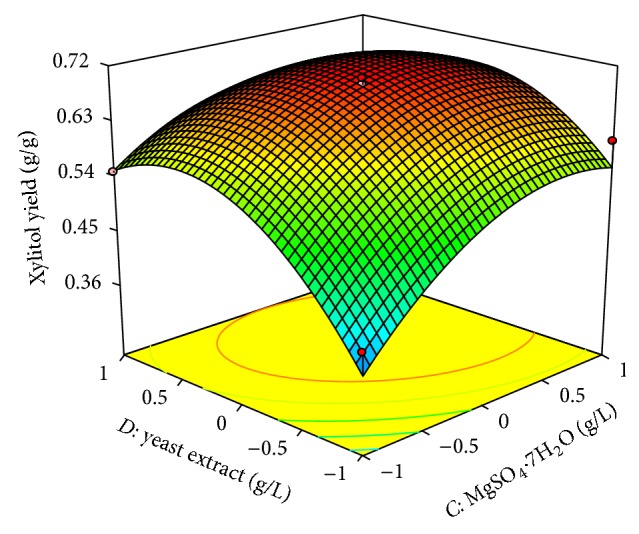
3D plot showing the effect of MgSO_4_·7H_2_O and yeast extract on xylitol yield.

In both designs the interaction effects of variables on xylitol production were studied by plotting 3D surface curves against any two independent variables, while keeping another variable at its central (0) level. The 3D curves of the calculated response (xylitol yield) and contour plots from the interactions between the variables were obtained.  Figure 2 shows the dependency of xylitol on peptone and xylose. The xylitol production increased with increase in peptone to about 6 g/L, and thereafter xylitol production decreased with further increase in peptone. The same trend was observed in Figures 3 and 4. This evidence from above figures shows the dependency of xylose, MgSO_4_·7H_2_O, yeast extract on xylitol production. The optimal operation conditions of peptone, xylose, MgSO_4_·7H_2_O, and yeast extract for maximum xylitol production were determined by response surface analysis and also estimated by regression equation. The predicted results were shown in Table 3. The predicted values from the regression equation closely agreed with that obtained from experimental values.

**Figure 5:**
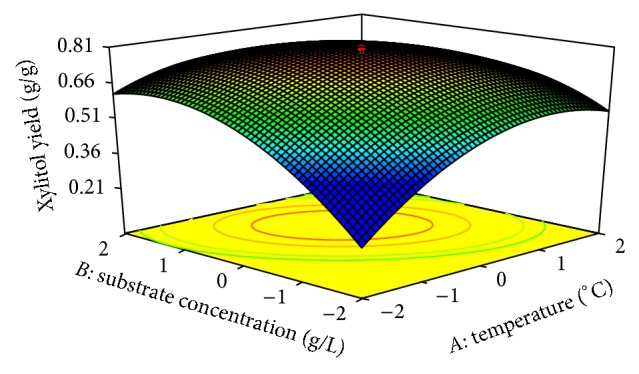
3D plot showing the effect of temperature and substrate concentration on xylitol yield.

**Figure 6:**
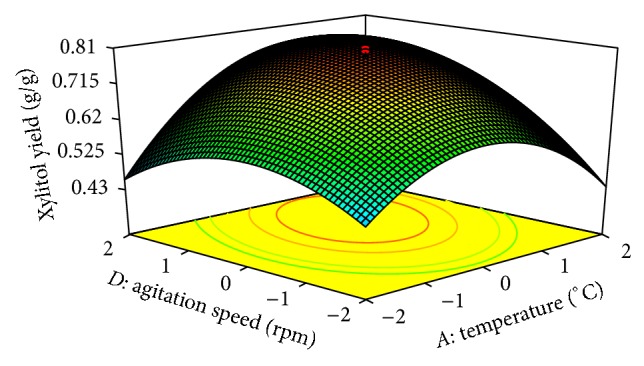
3D plot showing the effect of temperature and agitation speed on xylitol yield.

In CCD Figure 5 shows the dependency of xylitol on temperature and substrate concentration. The xylitol production increased with increase in temperature to about 36°C, and thereafter xylitol production decreased with further increase in temperature. The same trend was observed in Figure 6 and figures of other variables. This evidance from above figures shows the dependency of pH, substrate concentration, agitation speed, and inoculum size on xylitol production. The optimal operation conditions of temperature, substrate concentration, pH, agitation speed, and inoculum size for maximum xylitol production were determined by response surface analysis and also estimated by regression equation. The predicted results were shown in Table 6. The predicted values from the regression equation closely agreed with that obtained from experimental values.

### 3.1. Validation of the Experimental Model

Validation of the experimental model was tested by carrying out the batch experiment under optimal operation conditions which are (g/L): peptone: 6.03, xylose: 10.62, MgSO_4_·7H_2_O: 1.39, yeast extract: 4.66 established by the regression model. Under optimal process variables levels are temperature (36.56°C), pH (7.27), substrate concentration (3.55 g/L), inoculum size (3.69 mL), and agitation speed (194.44 rpm). Four repeated experiments were performed and the results are compared. The xylitol production (0.80 g/g) obtained from experiments was very close to the actual response (0.78 g/g) predicted by the regression model, which proved the validity of the model.

## 4. Conclusion

In this work, Plackett-Burman design was used to test the relative importance of medium components on xylitol production. Among the variables, peptone, xylose, MgSO_4_·7H_2_O, and yeast extract were found to be the most significant variables. From further optimization studies the optimized values of the nutrients for xylitol production were as follows (g/L): peptone: 6.03, xylose: 10.62, MgSO_4_·7H_2_O: 1.39, and yeast extract: 4.66. Then the influence of various process variables, namely, temperature, pH, substrate concentration, agitation speed, and inoculum size on the xylitol production was evaluated by CCD. The optimum levels of process variables are temperature (36.56°C), pH (7.27), substrate concentration (3.55 g/L), inoculum size (3.69 mL), and agitation speed (194.44 rpm). This study showed that the corncob is a good source for the production of xylitol. Using the optimized conditions, the xylitol yield reaches 0.80 g/g. The results show a close concordance between the expected and obtained production level. 

## Figures and Tables

**Table 1:** Plackett-Burman experimental design for nine variables.

Run order	*A*	*B*	*C*	*D*	*E*	*F*	*G*	*H*	*I*	Xylitol yield (g/g)
1	−1	1	−1	−1	−1	1	1	1	−1	0.47
2	1	−1	−1	−1	1	1	1	−1	1	0.34
3	1	1	−1	1	1	−1	1	−1	−1	0.44
4	−1	−1	−1	−1	−1	−1	−1	−1	−1	0.35
5	−1	−1	−1	1	1	1	−1	1	1	0.26
6	−1	−1	1	1	1	−1	1	1	−1	0.50
7	1	1	1	−1	1	1	−1	1	−1	0.49
8	−1	1	1	−1	1	−1	−1	−1	1	0.48
9	1	−1	1	−1	−1	−1	1	1	1	0.59
10	1	1	−1	1	−1	−1	−1	1	1	0.45
11	−1	1	1	1	−1	1	1	−1	1	0.69
12	1	−1	1	1	−1	1	−1	−1	−1	0.65

**Table 2:** Ranges of variables used in Box-Behnken design.

S. no	Variables	Code	Levels (g/L)		
			−1	0	1
1	Peptone	*A*	3	5	7
2	Xylose	*B*	8	10	12
3	MgSO_4_·7H_2_O	*C*	1	2	3
4	Yeast extract	*D*	2	4	6

**Table 3:** Box-Behnken design in coded levels with xylitol yield as response.

Runs	*A*	*B*	*C*	*D*	Xylitol Yield (g/g)	
					Experimental	Predicted
1	0	−1	1	0	0.44	0.47
2	0	1	0	−1	0.30	0.32
3	0	0	1	1	0.59	0.59
4	1	0	1	0	0.62	0.64
5	0	0	0	0	0.70	0.69
6	0	1	0	1	0.55	0.58
7	−1	0	0	1	0.50	0.50
8	−1	1	0	0	0.34	0.33
9	0	0	1	−1	0.60	0.56
10	0	−1	−1	0	0.39	0.41
11	−1	0	0	−1	0.36	0.41
12	0	0	0	0	0.70	0.69
13	0	0	−1	−1	0.40	0.36
14	−1	−1	0	0	0.51	0.48
15	−1	0	1	0	0.55	0.54
16	−1	0	−1	0	0.43	0.42
17	0	0	−1	1	0.55	0.55
18	1	0	0	1	0.66	0.62
19	1	0	0	−1	0.48	0.49
20	1	1	0	0	0.63	0.62
21	0	0	0	0	0.69	0.69
22	1	−1	0	0	0.43	0.40
23	0	1	1	0	0.58	0.57
24	1	0	−1	0	0.51	0.53
25	0	−1	0	1	0.39	0.39
26	0	1	−1	0	0.42	0.40
27	0	−1	0	−1	0.45	0.44
28	0	0	0	0	0.70	0.69
29	0	0	0	0	0.70	0.69

**Table 4:** Analyses of variance (ANOVA) for response surface quadratic model for the production of xylitol using Box-Behnker design.

Source	Sum of square	df	Mean square value	*F* value	*P* value
Model	0.40	14	0.028	26.29	<0.0001
*A*-peptone	0.034	1	0.034	31.67	<0.0001
*B*-xylose	3.675*E* − 003	1	3.675*E* − 003	3.41	0.0861
*C*-MgSO_4_ · 7H_2_O	0.039	1	0.039	35.75	<0.0001
*D*-yeast extract	0.035	1	0.035	32.67	<0.0001
*AB*	0.034	1	0.034	31.76	<0.0001
*AC*	2.500*E* − 005	1	2.500*E* − 005	0.023	0.8811
*AD*	4.000*E* − 004	1	4.000*E* − 004	0.37	0.5521
*BC*	3.025*E* − 003	1	3.025*E* − 003	2.81	0.1160
*BD*	0.024	1	0.024	22.29	0.0003
*CD*	6.400*E* − 003	1	6.400*E* − 003	5.94	0.0288
*A* ^2^	0.045	1	0.045	41.63	<0.0001
*B* ^2^	0.16	1	0.16	148.20	<0.0001
*C* ^2^	0.037	1	0.037	34.46	<0.0001
*D* ^2^	0.074	1	0.074	68.80	<0.0001
Residual	0.015	14	1.078*E* − 003		
Lack of fit	0.015	10	1.501*E* − 003	75.04	0.0004
Pure error	8.000*E* − 005	4	2.000*E* − 005		
Cor total	0.41	28			

**Table 5:** Ranges of variables used in CCD.

S. no	Variables	Code	Levels				
			−2.37	−1	0	1	2.37
1	Temperature (°C)	*A*	20	25	30	35	40
2	Substrate concentration (g/L)	*B*	1	2	3	4	5
3	pH	*C*	6	6.5	7	7.5	8
4	Agitation speed (rpm)	*D*	50	100	150	200	250
5	Inoculum size (mL)	*E*	1	2	3	4	5

**Table 6:** Central composite design (CCD) in coded levels with xylitol yield as response.

Runs	*A*	*B*	*C*	*D*	*E*	Xylitol yield (g/g)	
						Experiment	Predicted
1	−2.37	0	0	0	0	0.55	0.50
2	−1	1	1	1	1	0.74	0.76
3	−1	−1	1	1	−1	0.47	0.53
4	0	0	0	0	0	0.80	0.78
5	1	1	1	1	−1	0.62	0.68
6	−1	1	1	−1	−1	0.60	0.61
7	0	0	0	0	0	0.81	0.78
8	1	1	−1	−1	−1	0.43	0.50
9	0	0	0	−2.37	0	0.61	0.57
10	−1	1	−1	1	−1	0.58	0.59
11	1	−1	1	1	1	0.74	0.74
12	1	1	1	1	1	0.80	0.79
13	0	0	−2.37	0	0	0.44	0.41
14	0	−2.37	0	0	0	0.47	0.46
15	−1	−1	−1	−1	1	0.45	0.47
16	0	0	0	0	−2.37	0.59	0.51
17	−1	1	−1	1	1	0.64	0.66
18	2.37	0	0	0	0	0.65	0.62
19	0	0	0	0	0	0.81	0.78
20	−1	−1	1	1	1	0.65	0.60
21	1	1	−1	1	1	0.68	0.70
22	−1	1	1	−1	1	0.65	0.71
23	−1	−1	−1	1	1	0.43	0.46
24	−1	1	−1	−1	−1	0.50	0.54
25	0	0	0	0	0	0.81	0.78
26	0	2.37	0	0	0	0.72	0.66
27	0	0	2.37	0	0	0.69	0.65
28	0	0	0	0	0	0.81	0.78
29	1	−1	1	−1	−1	0.57	0.59
30	−1	1	−1	−1	1	0.67	0.62
31	−1	−1	−1	1	−1	0.42	0.41
32	0	0	0	0	0	0.78	0.78
33	1	1	1	−1	1	0.67	0.66
34	0	0	0	0	0	0.73	0.78
35	0	0	0	0	2.37	0.68	0.68
36	−1	1	1	1	−1	0.67	0.67
37	0	0	0	2.37	0	0.74	0.71
38	−1	−1	1	−1	−1	0.53	0.53
39	1	−1	−1	1	−1	0.53	0.56
40	−1	−1	−1	−1	−1	0.40	0.42
41	1	−1	1	1	−1	0.66	0.66
42	1	1	−1	1	−1	0.65	0.62
43	1	1	1	−1	−1	0.58	0.56
44	1	−1	−1	1	1	0.63	0.62
45	1	−1	−1	−1	1	0.57	0.55
46	1	−1	1	−1	1	0.63	0.67
47	1	1	−1	−1	1	0.57	0.58
48	−1	−1	1	−1	1	0.61	0.60
49	0	0	0	0	0	0.65	0.77
50	1	−1	−1	−1	−1	0.51	0.50

**Table 7:** Analyses of variance (ANOVA) for response surface quadratic model for the production of xylitol using CCD.

Source	Sum of square	df	Mean square value	*F* value	*P* value
Model	0.63	20	0.031	15.58	<0.0001
*A* temperature (°C)	0.026	1	0.026	13.07	<0.0001
*B*-substrate concentration (g/L)	0.079	1	0.079	38.99	<0.0001
*C*-pH	0.10	1	0.10	51.74	<0.0001
*D*-agitation speed (rpm)	0.038	1	0.038	18.76	0.0002
*E*-inoculum size (mL)	0.061	1	0.061	30.26	<0.0001
*AB*	0.027	1	0.027	13.42	0.0010
*AC*	5.281*E* − 004	1	5.281*E* − 004	0.26	0.6125
*AD*	0.011	1	0.011	5.40	0.0273
*AE*	1.531*E* − 004	1	1.531*E* − 004	0.076	0.7847
*BC*	3.003*E* − 003	1	3.003*E* − 003	1.49	0.2319
*BD*	6.328*E* − 003	1	6.328*E* − 003	3.14	0.0868
*BE*	9.031*E* − 004	1	9.031*E* − 004	0.45	0.5084
*CD*	7.812*E* − 005	1	7.812*E* − 005	0.039	0.8452
*CE*	9.031*E* − 004	1	9.031*E* − 004	0.45	0.5084
*DE*	3.125*E* − 006	1	3.125*E* − 006	1.552*E* − 003	0.9688
*A* ^2^	0.088	1	0.088	43.81	<0.0001
*B* ^2^	0.092	1	0.092	45.78	<0.0001
*C* ^2^	0.12	1	0.12	58.49	<0.0001
*D* ^2^	0.039	1	0.039	19.48	<0.0001
*E* ^2^	0.063	1	0.063	31.25	<0.0001
Residual	0.058	29	2.014*E* − 003		
Lack of fit	0.053	22	2.400*E* − 003	3	0.0699
Pure error	5.600*E* − 003	7	8.000*E* − 004		
Cor total	0.69	49			

## References

[1] Manz U., Vanninen E., Voirol F., Food R. A. Xylitol: its properties and use as a sugar.

[2] Wang Y. M., van Eys J. (1981). Nutritional significance of fructose and sugar alcohols. *Annual Review of Nutrition*.

[3] Emidi A. (1978). Xylitol its properties and food application. *Food Technology*.

[4] Hyvonen L., Koivistoinen P., Voirol F. (1982). Food technological evaluation of xylitol. *Advances in Food Research*.

[5] Bar A., Nabors L. O., Gelardi L. (1986). Xylitol. *Alternative Sweeteners*.

[6] Aguirre-Zero O., Zero D. T., Proskin H. M. (1993). Effect of chewing xylitol chewing gum on salivary flow rate and the acidogenic potential of dental plaque. *Caries Research*.

[7] Makinen K. K. (1976). The sugar that prevents that tooth decay. *The Futurist*.

[8] Mikkola J.-P., Salmi T. (2001). Three-phase catalytic hydrogenation of xylose to xylitol—prolonging the catalyst activity by means of on-line ultrasonic treatment. *Catalysis Today*.

[9] Melaji A. J., Hamalainen L.

[10] Winkelhausen E., Kuzmanova S. (1998). Microbial conversion of D-xylose to xylitol. *Journal of Fermentation and Bioengineering*.

[11] Converti A., Dominguez J. M. (2001). Influence of temperature and pH on xylitol production from xylose by *Debaromyces hansenii*. *Biotechnology and Bioengineering*.

[12] Converti A., Perego P., Sordi A., Torre P. (2002). Effect of starting xylose concentration on the microaerobic metabolism of *Debaryomyces hansenii*: the use of carbon material balances. *Applied Biochemistry and Biotechnology A*.

[13] Domınguez J. M., Gong C. S., Tsao G. (1997). Production of xylitol from D-xylose by *Debaryomyces hansenii*. *Applied Biochemistry and Biotechnology*.

[14] Gırio F. M., Roseiro J. C., Sa-Machado P., Duarte-Reis A. R., Amaral-Collaco M. T. (1994). Effect of oxygen transfer rate on levels of key enzymes of xylose metabolism in *Debaryomyces hansenii*. *Enzyme and Microbial Technology*.

[15] Ling H., Cheng K., Ge J., Ping W. (2011). Statistical optimization of xylitol production from corncob hemicellulose hydrolysate by *Candida tropicalis* HDY-02. *New Biotechnology*.

[16] Inglett G. E. (1970). *Corn: Culture, Processing and Production*.

[17] Barl B., Biliaderis C. G., Murray E. D., MacGregor A. W. (1991). Combined chemical and enzymic treatments of corn husk lignocellulosic. *Journal of Science of Food Agriculture*.

[18] Olsson L., Hahn-Hägerdal B. (1996). Fermentation of lignocellulosic hydrolysates for ethanol production. *Enzyme and Microbial Technology*.

[19] Balan V., Bals B., Chundawat S. P. S., Marshall D., Dale B. E. (2009). Ligocellulosic biomass pretreatment using AFEX. *Methods in Molecular Biology*.

[20] Liaw W., Chen C., Chang W., Chen K. (2008). Xylitol production from rice straw hemicellulose hydrolyzate by polyacrylic hydrogel thin films with immobilized candida subtropicalis WF79. *Journal of Bioscience and Bioengineering*.

[21] Montgomery D. C. (2001). *Design and Analysis of Experiments*.

[22] Li W., Du W., Liu D. (2007). Optimization of whole cell-catalyzed methanolysis of soybean oil for biodiesel production using response surface methodology. *Journal of Molecular Catalysis B*.

[23] Naveena B. J., Altaf M., Bhadrayya K., Reddy G. (2005). Direct fermentation of starch to L(+) lactic acid in SSF by Lactobacillus amylophilus GV6 using wheat bran as support and substrate: medium optimization using RSM. *Process Biochemistry*.

[24] Noordin M. Y., Venkatesh V. C., Sharif S., Elting S., Abdullah A. (2004). Application of response surface methodology in describing the performance of coated carbide tools when turning AISI 1045 steel. *Journal of Materials Processing Technology*.

